# Phase Relations in a NaFeO_2_-SnO_2_ (0–50 mol.% SnO_2_) System and the Crystal Structure and Conductivity of Na_0.8_Fe_0.8_Sn_0.2_O_2_

**DOI:** 10.3390/ma15103612

**Published:** 2022-05-18

**Authors:** Georgiy S. Shekhtman, Elena A. Sherstobitova, Mariya S. Shchelkanova, Evgenia A. Ilyina

**Affiliations:** Institute of High Temperature Electrochemistry, Ural Branch, Russian Academy of Sciences, 20 Akademicheskaya St., 620990 Ekaterinburg, Russia; ela.sherstobitova@gmail.com (E.A.S.); shchelkanova.mariya@mail.ru (M.S.S.); ilyina@ihte.uran.ru (E.A.I.)

**Keywords:** NaFeO_2_-SnO_2_ system, layered O3-type structure, Na^+^-ion conductivity, two-dimensional migration map, the TOPOS program package

## Abstract

With the view of developing new materials for sodium and sodium-ion power sources, NaFeO_2_-SnO_2_ (0–50 mol.% SnO_2_) powders were synthesized using a solid state method, and their phase composition and crystal structure were studied. A phase of the Na_0.8_Fe_0.8_Sn_0.2_O_2_ composition with a layered rhombohedral structure of the α-NaFeO_2_ type was found when the tin dioxide content was 20 mol.%. The phase produced was of an O3 structural type. O3-type phases have sufficiently good performance when used as cathode materials in sodium-ion batteries and, moreover, often have a rather high sodium-cation conductivity. A two-dimensional migration map was built using Voronoi–Dirichlet partition and TOPOS software package. The sodium-ion conductivity of Na_0.8_Fe_0.8_Sn_0.2_O_2_ at room temperature was rated low (10^−8^ S × cm^−1^ at 20 °C), which may be the result of channels too narrow for Na^+^ migration. The results obtained show that the application of the compound studied in this work as a solid electrolyte in sodium power sources is unlikely. It is the potential use of Na_0.8_Fe_0.8_Sn_0.2_O_2_ as the active material of cathodes in Na and Na-ion power sources that presents practical interest.

## 1. Introduction

Sodium power sources in which Na-β-Al_2_O_3_ and NASICON were used as solid electrolytes were considered the most promising electrochemical energy storage solution in the 1980s and 1990s [1]. Sodium–sulfur batteries were successfully developed for electric vehicles [2], spacecraft [3], and other applications [4,5]. However, they were later replaced by power sources with lithium-containing anodes, since, in spite of a considerably higher price of lithium, its use substantially increases the energy efficiency of the power source [6,7]. The appearance of a great number of high-conductivity lithium-cation solid electrolytes has made a great contribution to the successful development of lithium and lithium-ion batteries [8,9,10,11].

However, lithium is a rare metal, its abundance in the Earth’s crust is low, and the possibilities of extending the use of lithium-ion batteries to larger devices, for example, electric vehicles, will be restricted by low lithium availability. Lithium production will inevitably move to poorer deposits in the near future, which will make lithium more expensive and later scarce. At the same time, reserves of cheap sodium are practically inexhaustible (e.g., the World Ocean) [12,13,14]. For this reason, recently, there has been a renewal of interest in sodium power sources and, consequently, an appearance of works dedicated to the investigation of new solid electrolytes with sodium-cation conductivity and new electrode materials [15,16,17,18,19].

Layered oxides based on Na_x_MO_2_ compounds [20,21], where M stands for a transition element (i.e., Ti, V, Cr, Mn, Fe, Co, and Ni) or a combination of two [22,23] or more [24] transition elements, are nowadays considered to be promising cathode materials for sodium-ion batteries. The structural properties of such compounds are described in detail in [25,26]. It is shown that in the crystal structure of such compounds, edge-sharing MO_6_ octahedra form (MO_2_)_n_ sheets between which sodium cations are inserted. Depending on the arrangement of the octahedral layers, the coordination of alkaline cations can be octahedral (O), prismatic (P), or tetrahedral (T). The number of layers within the unit cell can be different. For example, according to the denomination suggested in [25,26], structural type O3 means that alkaline cations are in octahedral coordination and the unit cell contains three layers in which they lie. O3-type phases have sufficiently good performance when used as cathode materials in sodium-ion batteries. Thus, for example, a battery with Na_0.8_Ni_0.6_Sb_0.4_O_2_ as its cathode active material delivers the capacity of 107.3 mAh/g in the voltage range of 2–4.5 V and exhibits a good capacity retention of 98.5 mAh/g after 100 cycles [22]. The Na(Mn_0.25_Fe_0.25_Co_0.25_Ni_0.25_)O_2_ cathode compound delivers an initial discharge capacity of 180 mAh/g and a specific energy density of 578 W/kg [24]. Many O3-type phases also have a rather high sodium-cation conductivity [23], which is important for cathode materials.

The layered low-temperature α-modification of NaFeO_2_ also belongs to the O3 structural type [26]. This circumstance, and also the low cost and availability of the starting materials, prompted research into the electrochemical properties of the α-modification of NaFeO_2_ when used as the active material of cathodes in sodium-ion power sources [27,28,29]. The initial results confirmed the promising outlook of α-NaFeO_2_-based cathodes: the power source with the α-NaFeO_2_-based cathode and sodium anode delivered a stable reversible capacity of 80–85 mAh/g at voltages below 3.4 V and good thermal stability [28]. However, at voltages above 3.5 V, α-NaFeO_2_ undergoes irreversible structural changes related to the migration of iron ions [27], as a result of which the capacity for sodium ions intercalation and, consequently, the reversibility of the battery deteriorates considerably. It has been shown that a partial substitution of iron ions for cations of other transition metals, such as Mn, Co, Ni [30], and Ti [29,31], improves the stability of α-NaFeO_2_-structured phases at elevated voltages. In addition, α-NaFeO_2_-structured solid solutions in a NaFeO_2_-TiO_2_ system have been reported to exhibit a rather high sodium-cation conductivity [32]. The present paper continues research into the electrical properties and crystal structure of the phases formed in NaFeO_2_—M^IV^O_2_ systems and deals with the study of phase ratios, structure, and conductivity in the NaFeO_2_-SnO_2_ system.

## 2. Materials and Methods

### 2.1. Sample Preparation

Fe_2_O_3_, α-modification, (analytical grade), SnO_2_ (analytical grade), and Na_2_CO_3_ (reagent grade), all REACHIM RF, were used as starting components for synthesizing the materials under investigation. Calculated amounts of the previously dried starting reagents were weighed (FX40-CJ analytical balance, Tokyo, Japan, BMI Surplus), within the accuracy of ±0.0001 g, and mixed by grinding with ethanol in a porcelain mortar. The obtained mixtures were then sintered in Al_2_O_3_ crucibles at 700 °C to decompose Na_2_CO_3_. Afterwards, the reaction mixtures were ground, pressed into pellets, and heated again. In the case of NaFeO_2_, the maximum temperature of synthesis was 700 °C, and the time was 24 h. In compositions containing SnO_2_, phase formation was accomplished at higher temperatures; therefore, they were synthesized at 1100–1150 °C for 16–20 h and homogenized after 8–10 h in the process. Sintered substances were crushed into the powder (particle size less than 0.05 mm) and pressed into disks, ~1 cm in diameter and 0.1–0.2 cm thick, to be further used in electrical resistance measurements. The pressed disks were sintered at 1100 °C for 4–6 h in the powder of the same composition to eliminate the possibility of the loss of sodium oxides owing to volatility at high temperatures. The open porosity of the resulting samples did not exceed 8%.

### 2.2. Characterization and Electrical Measurements

Phase and structural characterization of the samples during various stages of synthesis was carried out using the X-ray powder method with a Rigaku D/MAX-2200 VL/PC X-ray diffractometer (RIGAKU, Tokyo, Japan, monochromatic Cu *Kα*-radiation generated at 40 kW, 30 mA (*λ* = 1.54178 Å), 2*θ* = 10–80° stepwise with a 0.3 s counting time and a step of 0.02°). Jade 6.5 software (Materials Data Inc., Livermore, CA, USA) was used to calculate unit cell parameters. The errors in the cell parameter calculations did not exceed 0.02%. The structural parameters were refined by the Rietveld technique using the Full Prof program [33].

To determine the migration map of Na^+^ cations, we used the Voronoi–Dirichlet approach [34] implemented into the program package TOPOS [35].

The morphology and microstructure of the samples were examined by scanning electron microscopy (electron microscope TESCAN MIRA 3 LMU, Brno, Czech Republic).

Thermal analysis was carried out using a DSC 204 F1 Phoenix unit (NETZSCH, Selb, Germany). The measurement was conducted over the temperature range 25–1050 °C in air in Pt crucibles with a rate of heating of 10 °C/min. The results obtained were processed by means of NETZSCH Proteus software (STA 449 F1A 0030-M, Selb, Germany).

The electrical resistance of the samples was measured with Ag electrodes by analysis of the impedance spectrum obtained using Elins P-40x potentiostat-galvanostat (Elins, Zelenograd, Russia) with the FRA-24M module for electrochemical impedance measurements over the frequency range of 3 Hz–500 kHz. The electronic component was estimated by the DC method with gold electrodes at 20–40 mV.

## 3. Results and Discussion

### 3.1. XRD Analysis and Phase Relations

**Figure 1:**
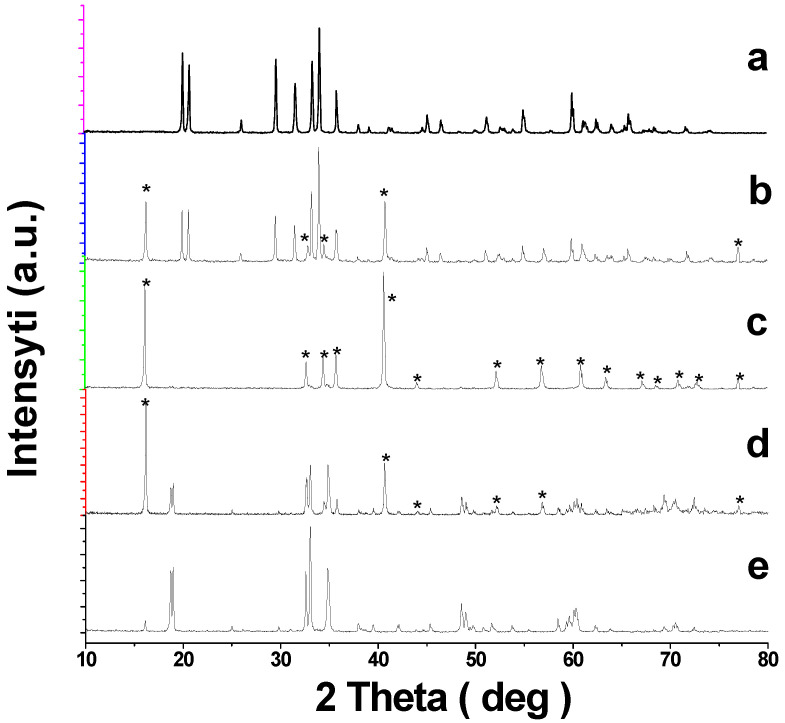
Powder XRD patterns for the samples of the NaFeO_2_—SnO_2_ system: (**a**) NaFeO_2_; (**b**) 5; (**c**) 20; (**d**) 35 mol.% SnO_2_; (**e**) NaFeSnO_4_. * Designates the lines for the phases with a rhombohedral structure.

Sodium ferrite (NaFeO_2_) exists in three crystal modifications: α, β, and γ [36]. The low-temperature α-modification of NaFeO_2_ undergoes an irreversible transition into the β-form at 760 °C [37], and the latter turns into the γ-form when heated to ~1000 °C [38,39]. It is known that the structure of NaFeO_2_ synthesized at temperatures below 760 °C, to a large extent, depends on the nature and structure of the starting components, especially iron oxide [36]. For instance, heating a mixture of NaOH and α-Fe_2_O_3_ at 200 °C yields α-NaFeO_2_, while α-Fe_2_O_3_ and Na_2_CO_3_ used as starting components result in β-NaFeO_2_, even if the synthesis is below 760 °C [37]. The α-form of sodium ferrite is obtained through sintering the mixture of Fe_3_O_4_ and Na_2_CO_3_ [29], and also Fe_3_O_4_ and Na_2_O_2_ [27] at 650 °C. In [31], sodium oxalate and Fe_2_O_3_ were used to produce α-NaFeO_2_, and the modification of the second starting component is not specified. In this work, we used Na_2_CO_3_ and α-Fe_2_O_3_ to synthesize NaFeO_2_. According to XRD, sintering at 700 °C yields β-modification of NaFeO_2_, PDF2-76-0243 (Figure 1a), which is in line with the data in [36].

According to [37], β-NaFeO_2_ should be metastable below 760 °C; however, treatment at 700 °C for 600 h did not bring about a change in the structure [36].

When tin dioxide was added to NaFeO_2_, even in relatively small amounts (~5 mol.%), extra reflections of a rhombohedral phase with a structure close to α-NaFeO_2_ appeared on the XRD patterns together with β-NaFeO_2_ reflections (Figure 1b). These reflections of the rhombohedral phase grew in intensity as the tin content increased, and when it reached 20 mol.%, the lines of β-NaFeO_2_ disappeared, and the XRD pattern for the Na_0.8_Fe_0.8_Sn_0.2_O_2_ sample contained only the reflections of the rhombohedral phase (Figure 1c). With a further increase in the content of SnO_2_ reflections of NaFeSnO_4_, PDF2-73-0425 existed on the X-ray patterns of the samples (Figure 1d,e). Thus, unlike a similar titanium-containing system [31], NaFeO_2_-SnO_2_ system was not characterized by a wide region of solid solutions with an O3-type structure.

### 3.2. Morphology Study and Thermal Analysis

**Figure 2:**
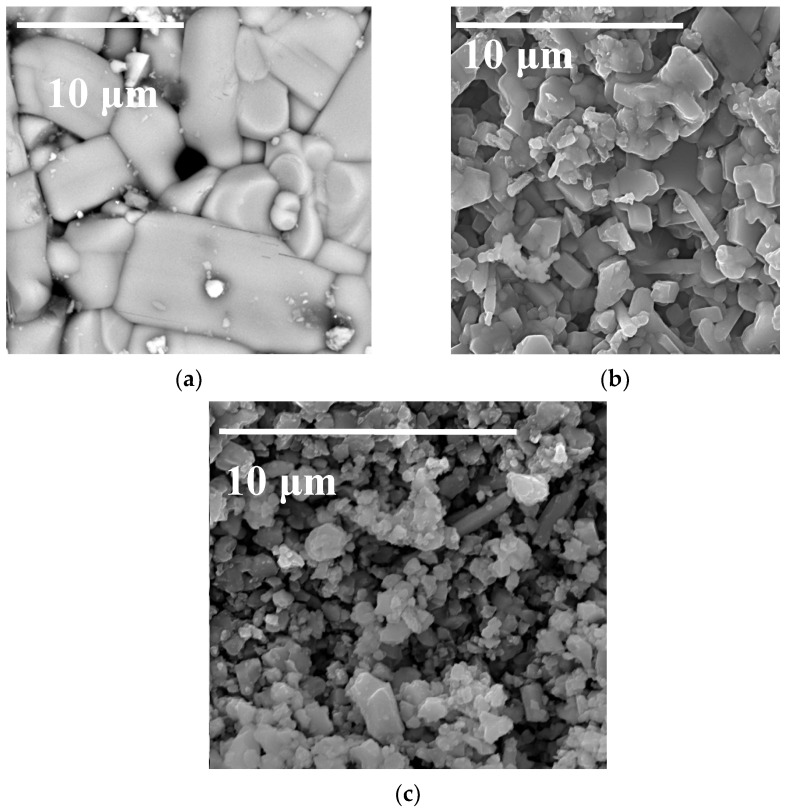
SEM image of the surface of the sintered Na_0.8_Fe_0.8_Sn_0.2_O_2_ pellets in the NaFeO_2_-SnO_2_ system: (**a**) 5; (**b**) 20 (Na_0.8_Fe_0.8_Sn_0.2_O_2_); (**c**) 35 mol.% SnO_2_.

Figure 2 contains SEM images of the surface for the number of sintered pellets in the NaFeO_2_-SnO_2_ system. One can see that the microstructure of the sample that contained 5 mol.% SnO_2_ (Figure 2a) was not homogeneous; it had the form of grains 1–10 µm in size and inclusions of the second phase (NaFeO_2_-SnO_2_) can be seen. The structure of the sample containing 20 mol.% SnO_2_ (Figure 2b) was more homogeneous and consisted of grains 1–2 μm in size. The sample had a rather high density, though some pores can be seen. The sample containing 35 mol.% SnO_2_ had a fine crystalline structure.

**Figure 3:**
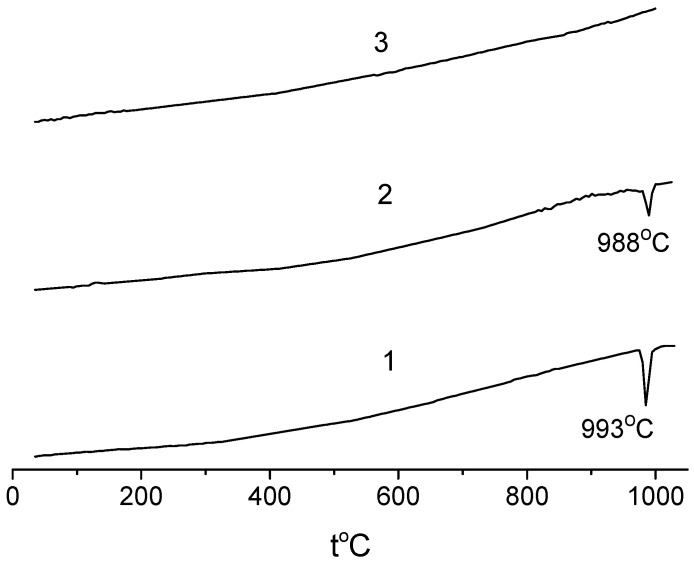
DSC curves for samples of the NaFeO_2_—SnO_2_ system: (1) 5; (2) 10; (3) 20 mol.% SnO_2_.

Figure 3 shows the DSC curves for the samples with 5, 10, and 20 mol.% SnO_2_. As one can see, there were peaks at approximately1000 °C on the first two curves, while the third curve, which corresponds to the Na_0.8_Fe_0.8_Sn_0.2_O_2_ sample, contained no thermal effects. The temperature of the peaks on curves 1 and 2 was close to the ones in [38,39], which were ascribed to the β → γ transition in NaFeO_2_. Figure 1 indicates that the content of β-phase in the samples decreased with an increase in the content of SnO_2_. Returning to Figure 3, it is plain to see that the intensity of the thermal effect on the DSC curves also decreased with an increase in the tin content (Figure 3, curves 1 and 2). The sample with 20 mol.% SnO_2_ contained no β-phase, and there were no peaks on the DSC curve of this sample (Figure 3, curve 3). Thus, there is every reason to believe that the thermal effects on the DSC curves (Figure 3) corresponded to the β → γ transition, while the α-solid solutions were stable across the whole temperature range studied.

The higher stability of α-solid solutions in the NaFeO_2_-SnO_2_ system, generated by the introduction and growing increase in the tin oxide content, may be explained as follows: the α-modification of NaFeO_2_ has a rhombohedral rock-salt structure, where the Fe^3+^ ions occupy octahedral positions in the cubic closest packed oxygen layers [36], while the orthorhombic Pbn2_1_ structure of β-NaFeO_2_ is derived from the wurtzite structure, in which Fe cations occupy tetrahedral positions [38]. According to [40], Ti^4+^ ions often prefer octahedral coordination to the tetrahedral one. This fact is considered by the authors of [31] to be the reason for the increased stability of the α-form of NaFeO_2_ when the ions of iron are substituted for titanium ions, which is manifested in the growing temperature of the α → β transition. The same reason may be true in the situation when iron is replaced by tin, especially since the radius of Sn^4+^ ion is bigger than the radius of Fe^3+^ (0.69 and 0.63 Å, respectively, for the tetrahedral coordination [41]); therefore, even small amounts of added tin induce a partial transition of β-NaFeO_2_ into α-modification, while the α → β transition does not take place with increasing temperature.

### 3.3. Crystal Structure of Na_0.8_Fe_0.8_Sn_0.2_O_2_

**Figure 4:**
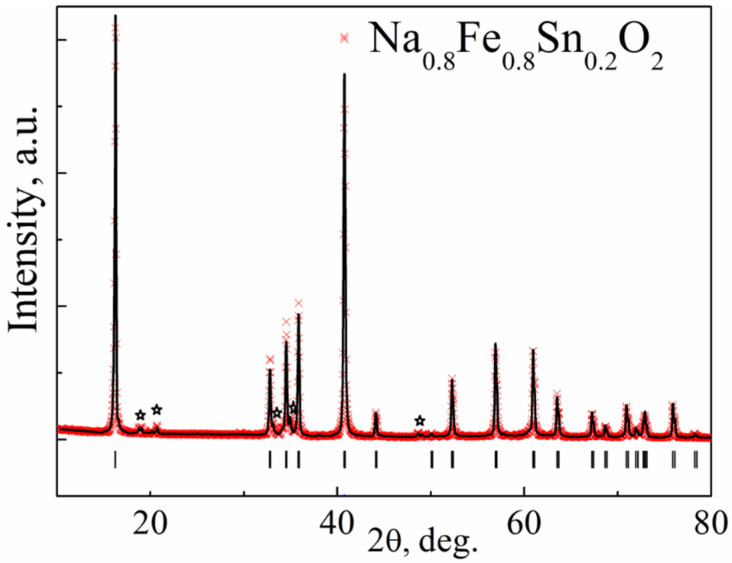
The result of the Rietveld refinement of the X-ray diffraction pattern of Na_0.8_Fe_0.8_Sn_0.2_O_2_. The experimental profile is represented by the red symbols (×), while the calculated profile is represented by the black line. Vertical bars denote Bragg peak positions of the main rhombohedral phase (PDF2-20-1115). Asterisks mark (**✩**) the monoclinic impurity phase, as it was reported in Reference [37].

Figure 4 shows Rietveld refinement using the X-ray pattern of Na_0.8_Fe_0.8_Sn_0.2_O_2_.

**Figure 5:**
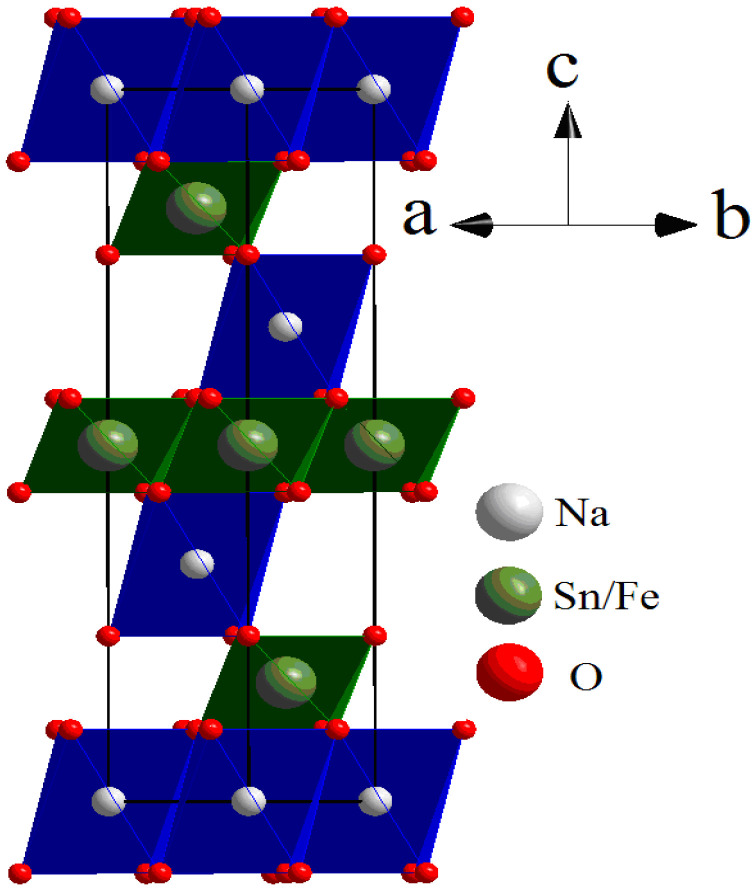
Rhombohedral crystal structure of Na_0.8_Fe_0.8_Sn_0.2_O_2_. Red, white, and green spheres represent oxygen, sodium, and Fe/Sn atoms, respectively.

The diffraction pattern contained highly intensive reflections from the main phase with a rhombohedral unit cell and s. g. R-3m (No.166, PDF2-20-1115). The crystal structure of Na_0.8_Fe_0.8_Sn_0.2_O_2_ can be represented as layers of (Fe,Sn)O_6_ octahedra alternating with layers of NaO_6_ octahedra. The atoms of Na occupy 3b sites in their layer; the atoms of O occupy 6c sites. The atoms of Sn and Fe were statistically mixed in 3a sites. The crystal structure of Na_0.8_Fe_0.8_Sn_0.2_O_2_ is given in Figure 5. The refined structural parameters for the rhombohedral O3-type phase are shown in Table 1.

The X-ray diffraction pattern of the Na_0.8_Fe_0.8_Sn_0.2_O_2_ sample contained additional broad reflections of low intensity at 2*θ* ~ 20° (Figure 4), which were not connected with the main phase of the α-NaFeO_2_ type and were apparently determined by the honeycomb-like ordering of the Fe and Sn ions within the layer. Broadening of the reflections can be caused either by a nonideal ordering of Fe and Sn ions (the presence of a short-range order) or by a stacking fault [42]. The phase with Fe and Sn ion ordering can be satisfactorily described as a monoclinic unit cell, s. g. C2/m, and the structural model suggested for Na_0.89_Zn_1/3_Ir_2/3_O_2_ [43].

### 3.4. Conductivity Study

**Figure 6:**
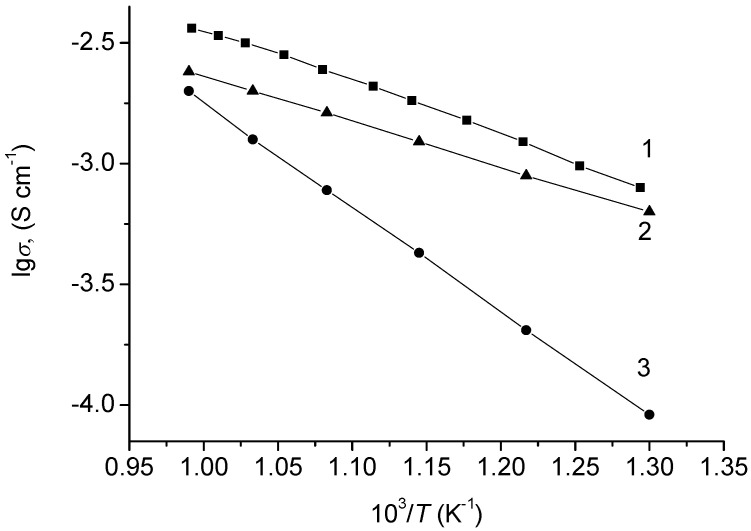
Temperature dependences of NaFeO_2_ conductivity: (1) total; (2) ionic; (3) electronic conductivity.

Undoped β-NaFeO_2_ had a mixed electronic-ionic conductivity, where at 600–700 °C the electronic and ionic components had close values, and at lower temperatures ionic conductivity prevailed (Figure 6).

The sodium-cation character of the ionic component of sodium ferrite conductivity was confirmed in [23]. According to the obtained results, the conductivity of NaFeSnO_4_ was 10^−4^–10^−3^ S × cm^−1^ over the 500–700 °C interval and was mostly ionic in character.

**Figure 7:**
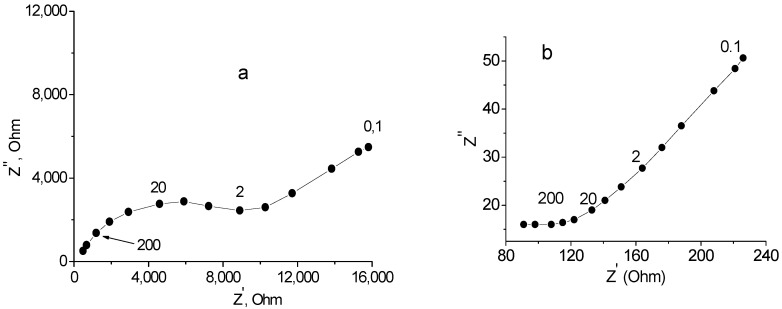
Impedance spectra for Ag|Na_0.8_Fe_0.8_Sn_0.2_O_2_|Ag cell: (**a**) 182; (**b**) 368 °C. Numbers above the curves represent frequencies (kHz).

The conductivity of the Na_0.8_Fe_0.8_Sn_0.2_O_2_ sample was found by analysis of the impedance spectrum over the temperature range 20–370 °C. The typical impedance spectra are shown in Figure 7. In the low-temperature region, the spectra had the form of a low-frequency tail and a semicircular arc with a shifted center (Figure 7a). The total resistance, R_b+gb_, of the samples under investigation was found as the values corresponding to the points of intersection between the semicircle and the Z’-axis. Above 250 °C, the spectra contained only a low-frequency tail (Figure 7b); in this case, the resistance of the sample was found by extrapolation of the low-frequency linear portion onto the real axis.

**Figure 8:**
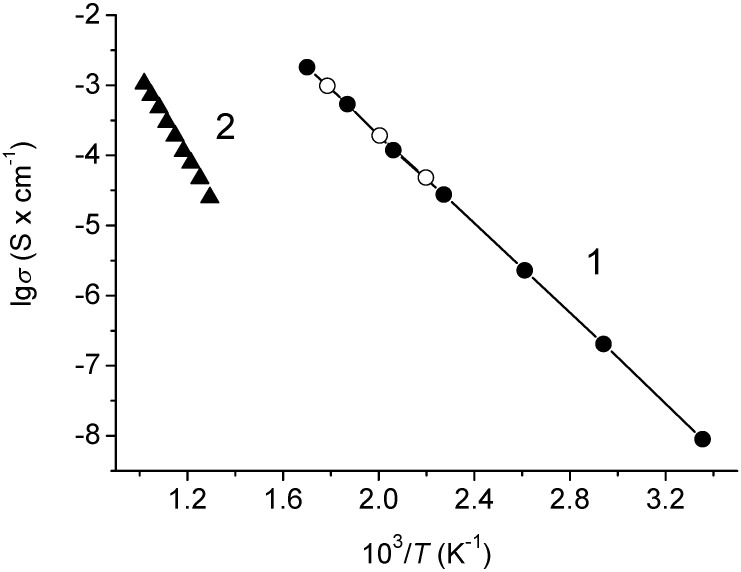
Temperature dependences of electroconductivity for Na_0.8_Fe_0.8_Sn_0.2_O_2_: (1) total conductivity; solid circles—heating, open circles—cooling; (2) electronic conductivity.

The temperature dependences of total and electronic conductivities for Na_0.8_Fe_0.8_Sn_0.2_O_2_ are given in Figure 8.

One can see that the dependence of the total conductivity in the lg*σ*—1/*T* coordinates was linear across the studied temperature range (Figure 8, line 1). The activation energy for conduction was 64.2 ± 0.43 kJ × mol^−1^. The electronic conductivity at 500 °C was approximately 10^−5^ S × cm^−1^ and decreased fast with the decreasing temperature (Figure 8, line 2). Thus, in the low-temperature region, Na_0.8_Fe_0.8_Sn_0.2_O_2_ was a practically unipolar sodium-cation conductor.

### 3.5. Exploration of the Migration Map of Na^+^ Cations

**Figure 9:**
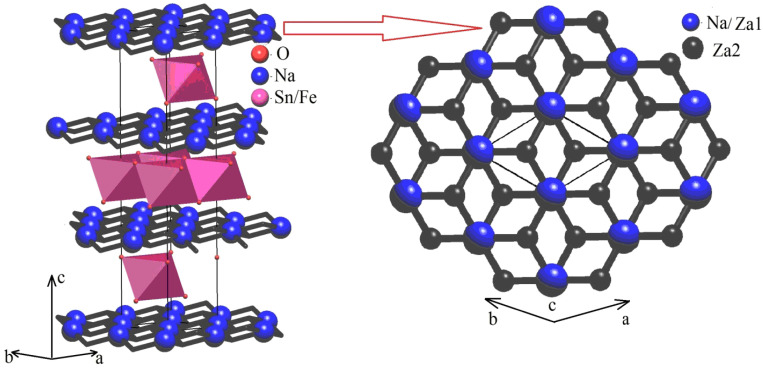
Migration map (**left**) and 2D migration layer (**right**) for rhombohedral Na_0.8_Fe_0.8_Sn_0.2_O_2_; large blue spheres—Na^+^ ions coincide with Za1 significant voids, black spheres—Za2 significant voids in the center of the vacant tetrahedral interstitial site, and black lines—elementary migration channels, Sn/FeO6 octahedra are pink.

In order to explore the Na^+^ cations migration map (i.e., network of cations migration pathways within the crystal structure framework of the rhombohedral structure of Na_0.8_Fe_0.8_Sn_0.2_O_2_), we used the Voronoi–Dirichlet approach [35] implemented into the program package TOPOS [44]. The elementary voids and channels were searched using the Voronoi–Dirichlet polyhedra partitioning space as it was described in detail in [35]. According to this approach, vertices of the Voronoi–Dirichletpolyhedral represent centers of the elementary voids, while the edges of the Voronoi–Dirichletpolyhedral represent the elementary channel lines connecting the centers of nearby voids. In order to build the ion migration map, all elementary channels are supposed to be sorted out by using significance criteria [34]. Only “significant” voids and channels were involved in the construction of the ion migration map. According to [16], a void was considered to be significant for Na^+^ cations in an oxygen environment when the characteristic size of the void was Rsd ≥ (1.523 ± 0.040) Å. An elementary channel was considered to be significant when the characteristic size of the channel was Rsd ≥ 2.083 Å. The coordinates of the first void, ZA1, coincided with the position of the Na atoms in the NaO_6_ octahedron. The coordinates of the second void, ZA2, almost fully coincided with the coordinates of the center of the vacant tetrahedral interstitial site (2/3 1/3 0.6969) in the NaO layer. The centers of the ZA1 and ZA2 voids were connected by an elementary channel of conductivity with the radius *r* = 2.048 Å. Using the discovered ZA1 and ZA2 voids and one elementary channel of conductivity, one can build a 2D migration map in which migration channels connect the centers of NaO_6_ octahedra with the vacant tetrahedron through the shared edge (see Figure 9).

One can easily notice that the radius of the elementary migration channel in the lattice of the investigated compound (2.048 Å) was rather smaller compared to the critical channel diameter for Na^+^ cations in oxide compounds (2.083 Å). Nevertheless, this is not the reason to exclude such channels from the migration map. Geometrical analysis and, in particular, Voronoi–Dirichlet partition, characterized the static model of a crystal structure. Migration of the charge carriers caused the crystal lattice to undergo considerable distortions and, as a result, mobile ion transfer becomes possible through the channels that are smaller than the critical size when the structure is static [45]. Insufficient width of migration channels, nevertheless, may be one of the reasons why sodium-cation conductivity of Na_0.8_Fe_0.8_Sn_0.2_O_2_ at low temperatures is rather unremarkable (10^−8^ S × cm^−1^ at 20 °C, Figure 8, line 1).

Previously the studies of NaFeO_2_-TiO_2_ system reported high sodium-cation conductivity of the obtained solid solutions, which gave reason to consider their potential application as solid electrolytes in sodium power sources [32]. The conductivity values for the Na_0.8_Fe_0.8_Sn_0.2_O_2_ phase obtained in the present work were considerably lower compared with the ones in [32]. It should also be remarked that in reducing environments, Fe^3+^ can be easily reduced to Fe^2+^. For instance, the Na_4_FeO_3_ compound may form in contact with metallic sodium [46]. Sn^4+^ ions can also be reduced to Sn^2+^. Both of these factors will lead to a sharp increase in electronic conductivity; therefore, it is highly unlikely that the compound studied in this work will be used as a solid electrolyte. Na_0.8_Fe_0.8_Sn_0.2_O_2_ is of much practical interest as the potential cathode material in Na^+^ power sources. Additional electrochemical studies are required to confirm this possibility.

## 4. Conclusions

Samples of the NaFeO_2_-SnO_2_ (0–50 mol.% SnO_2_) system were obtained by solid state synthesis for the first time, and their phase composition was studied. A novel phase having a Na_0.8_Fe_0.8_Sn_0.2_O_2_ composition and an O3-type rhombohedral layered lattice was found, and Rietveld refinement of its crystal structure was performed. The conductivity of the Na_0.8_Fe_0.8_Sn_0.2_O_2_ was of the sodium-cation type and at a room temperature of 10^−8^ S × cm^−1^ with the activation energy of 64.2 ± 0.43 kJ·mol^−1^. A migration map was built using the Voronoi–Dirichlet approach. The radius of the elementary migration channel in the Na_0.8_Fe_0.8_Sn_0.2_O_2_ lattice was slightly smaller than the size critical for Na^+^ cations in oxide compounds, which may be the reason why the sodium-cation conductivity of the Na_0.8_Fe_0.8_Sn_0.2_O_2_wasrather low at low temperatures. The obtained results show that the application of the compound studied in this work as a solid electrolyte in sodium power sources is unlikely. It is the potential use of Na_0.8_Fe_0.8_Sn_0.2_O_2_ as the active material of cathodes in Na and Na-ion power sources that presents practical interest.

## Figures and Tables

**Table 1:** Refined structural parameters for Na_0.8_Fe_0.8_Sn_0.2_O_2_, rhombohedral cell, s. g. R-3 m (No. 166), a = 3.0381(6), Å,c = 16.3895(4) Å.

Refined Structural Parameters	Atom		
	Na (3b)	Fe/Sn (3a)	O (6c)
Atom coordinates	001/2	000	00z
Thermal parameters, Å^2^	1.31(4)	0.71(2)	0.52(4)
χ^2^	3.651		
Bragg R-factor	2.91		

## Data Availability

The data presented in this study are available upon request from the corresponding author.
